# Machine Learning-Based Facial Beauty Prediction and Analysis of Frontal Facial Images Using Facial Landmarks and Traditional Image Descriptors

**DOI:** 10.1155/2021/4423407

**Published:** 2021-08-25

**Authors:** Tharun J. Iyer, Rahul K., Ruban Nersisson, Zhemin Zhuang, Alex Noel Joseph Raj, Imthiaz Refayee

**Affiliations:** ^1^School of Electrical Engineering, Vellore Institute of Technology, Vellore 632014, India; ^2^Department of Electronic Engineering, Shantou University, Shantou 515063, China; ^3^Chairman NB Multispecialty Dental Hospital, Chennai, India

## Abstract

The beauty industry has seen rapid growth in multiple countries and due to its applications in entertainment, the analysis and assessment of facial attractiveness have received attention from scientists, physicians, and artists because of digital media, plastic surgery, and cosmetics. An analysis of techniques is used in the assessment of facial beauty that considers facial ratios and facial qualities as elements to predict facial beauty. Here, the facial landmarks are extracted to calculate facial ratios according to Golden Ratios and Symmetry Ratios, and an ablation study is performed to find the best performing feature set from extracted ratios. Subsequently, Gray Level Covariance Matrix (GLCM), Hu's Moments, and Color Histograms in the HSV space are extracted as texture, shape, and color features, respectively. Another ablation study is performed to find out which feature performs the best when concatenated with the facial landmarks. Experimental results show that the concatenation of primary facial characteristics with facial landmarks improved the prediction score of facial beauty. Four models are trained, K-Nearest Neighbors (KNN), Linear Regression (LR), Random Forest (RF), and Artificial Neural Network (ANN) on a dataset of 5500 frontal facial images, and amongst them, KNN performs the best for the concatenated features achieving a Pearson's Correlation Coefficient of 0.7836 and a Mean Squared Error of 0.0963. Our analysis also provides us with insights into how different machine learning models can understand the concept of facial beauty.

## 1. Introduction

Facial beauty has long been a topic of intellectual discussion and its various attributes have been researched upon and studied. In medieval times, renaissance painters used unique ratios named “The Golden Ratios” to represent through paintings what the perfectly shaped human face would look like [1]. The Golden Ratios are ratios based on the value of 1.6, which was considered by the Greeks to be a perfect number. Many examples exist in architecture, too, from the Greek Empire, where the golden ratio was used in buildings and pantheons. This golden ratio was applied to facial beauty, where different facial ratios were calculated and compared against the value of 1.6. Although facial attractiveness can be debated on its objectivity or subjectivity, recent empirical results support the idea that attractiveness is objective and quantifiable, which is achieved by measuring cross-cultural differences [2], brain activity [3], and cognitive psychology [4]. Studies from medicine and psychology have also concluded that aesthetic features like facial averageness [5] and symmetry [6] are important when assessing attractiveness. Facial skin colors and texture also significantly contribute towards facial attractiveness [7, 8] and have been included in assessments of facial attractiveness [9, 10]. Moreover, research has shown that attractive faces follow defined ratios of facial proportions, such as neoclassical canons [11] and the golden ratio [12], which is considered the golden standard for beautiful faces since ancient times by artists, physicians, and cosmetic surgeons [13]. In machine learning, several methods have been proposed to assess facial attractiveness by using these facial features. However, due to less efficient feature extraction methods and the inability to combine various features together, this method has not been worked upon, even though this approach may close the gap between human and machine performance. Hence, there is a need for an efficient technique of facial beauty assessment from a machine's perspective. In this paper, a technique for assessing facial beauty based on facial proportion factors is developed, which is widely believed to be the gold standard for facial beauty.

Research conducted in Psychology and Biology settles the problem by making a hypothesis of which facial features contribute to attractiveness. Various features like sexual dimorphism, averageness, and symmetry influence the perception of beauty. Jones and Jaeger [10] showed that women appear more attractive based on these three features. Thornhill and Gangestad [14] proved that there is a significant correlation between average faces and facial beauty, but the most attractive faces are not average. Facial symmetry, although, does increase with facial averageness, as stated in Grammer and Thornhill [15], which also supports the same idea from evolutionary biology studies. Moreover, sexual dimorphism is shown to play a significant role in assessing a person's facial beauty, as stated by Perrett et al. [16]. Sexual dimorphism is the presence of secondary sexual characteristics which appear during beauty. These secondary characteristics make people appear more masculine or feminine. Many studies provide evidence that masculinity and femininity provide more depth to a person's beauty than symmetry [10, 17, 18]. Intrinsic features of the face, such as facial texture and skin color, can also affect the perception of beauty. Many researchers have proposed a connection between facial beauty and healthy skin, which consequently proves that the health of facial skin might be a surface-level feature that influences beauty assessments. Fink et al. [7] assessed facial beauty through the use of human ratings from facial textures and skin color. Fink et al. [19] show that the health of facial skin is positively correlated with the attractiveness index of the face. Also, since facial attractiveness is affected by various factors, facial shape features and appearance have also been considered for facial beauty assessments. Kagian et al. [9] analyzed facial beauty depending on the shape and facial geometry. Rhazi et al. [20] proposed a method to predict facial beauty based on golden ratios calculated from the extracted feature corners. Schmid et al. [21] have proposed a model to calculate facial beauty based on golden ratios, symmetry, and neoclassical canons. The neoclassical canons are ratios used by medieval painters in their paintings to represent their understanding of human beauty. Dornaika et al. [22] developed a semisupervised face beauty prediction technique using a graph-based method with a public dataset. Though semisupervised techniques require fewer training images, their model accuracy entirely depends on the graph density, which affects the prediction results. Lin et al. [23] used Attribute aware CNN to predict the facial beauty, with SCUT-FPB5500 dataset and trained with powerful GPU support. Xiao et al. [24] developed Beauty3DFaceNet, which is comprised a deep CNN to predict the attractiveness of 3D faces. They collected the 3D point cloud and facial texture of the image to train their network which will output attractiveness score. Although the approach is promising, they are limited by the data available to collect the 3D point cloud and also the training requires extensive computing. Wei et al. [25] assessed facial symmetry and attractiveness based on SVM and linear regression using a predefined dataset. Also, they have developed a mobile app based on their concluded features which are useful for plastic surgeons to plan reconstructive facial surgeries. Tong et al. [26] investigated facial attractiveness using facial putative ratios and DNN by training with 4512 face images. The DNN model was trained using NVIDIA Tesla K40 GPU. Recent research takes advantage of CNN and DNN to predict facial attractiveness, which is computationally expensive and requires a large number of training and testing datasets. Since facial beauty prediction mainly depends on how effectively the facial features are analyzed, it is required to determine which features influence the most. Thus, by determining the effective features, facial beauty prediction could be achieved with less computationally expensive state-of-the-art machine learning techniques.

This paper aimed to predict the facial beauty of frontal images using machine learning techniques and traditional feature extraction methods. The proposed technique improves the performance of machine learning models on facial images through the fusion of facial landmarks and Texture, Color, and Shape Features. Nineteen facial ratios based on Golden Ratios and Symmetry are used in this paper. These facial ratios are fed as input to four regression-based machine learning models (Linear Regression, Random Forest, K-Nearest Neighbors, and an Artificial Neural Network) and trained to predict the beauty of the facial image. An Ablation Study is performed on the nineteen ratios to find out the best performing combination of ratios called the “Feature Set.” The Feature Set is concatenated with texture, color, and shape features (TCS Features). Also, another ablation study is performed to check the performance of each model in accurately predicting facial beauty. The ablation study is used to find out which of the TCS Features contributes to the prediction and how the model performance varies among each combination. The rest of the paper is organized as follows: At first, the steps to predict the facial beauty (extraction of the features from the dataset) are provided. Then, the discussion of various models used to predict facial beauty has been provided. Finally, the results of the implementation of various models and the best performing model are discussed.

## 2. Materials and Methods

**Figure 1:**
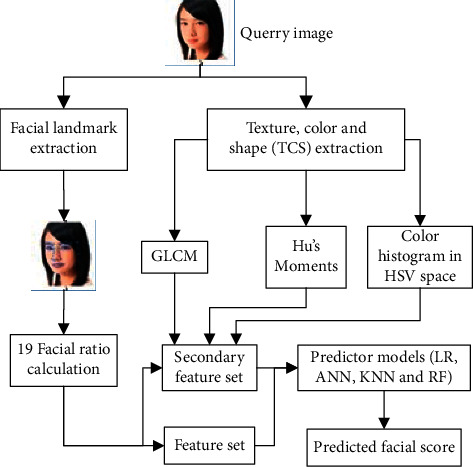
Block diagram of the analysis performed.

The following steps were used to predict the facial beauty score (Figure 1 represents the block diagram of the entire process):

- Extraction of facial landmarks, texture, color, and shape (TCS) features.
- Extraction of best performing facial landmark feature set.
- Ablation study of TCS Features.

### 2.1. Dataset Considered

The dataset used in this study is SCUT-FBP5500, a dataset that consists of 5500 images of Asian and Caucasian males and females [27], where the dimension of each image is 350×350 pixels. The dataset contains 5500 frontal, unoccluded faces aged from 15 to 60 with a neutral expression. It can be divided into four subsets with different races and genders, including 2000 Asian females, 2000 Asian males, 750 Caucasian females, and 750 Caucasian males. All the images are labeled with beauty scores ranging from 1–5 by a total of 60 volunteers aged from 18–27 (average 21.6), where the beauty score 5 means most attractive and a score of 1 or less means least attractive. The metrics used in this study to measure performance are Pearson's Correlation Coefficient (PC), Mean Absolute Error (MAE), Mean Squared Error (MSE), and *R*2 Score. All the models are trained on the same dataset using Python 3.7 software.

#### 2.1.1. Facial Landmark Localization

**Figure 2:**
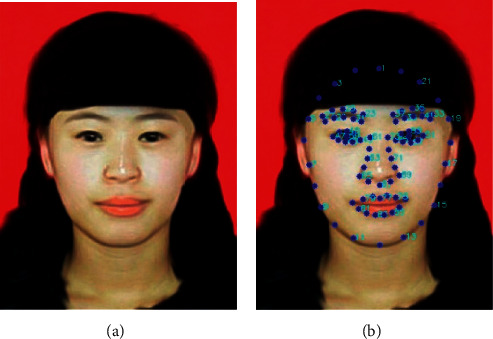
Sample image and its facial landmarks. (a) Sample image. (b) Facial landmarks of the sample image.

Facial ratios are calculated by measuring the distance between certain points on the face image. These points are called Facial landmarks, which are coordinates on the face image. The SCUT-FBP dataset contains predefined facial landmarks for all the 5500 images. Each image had a total of 86 landmarks that covered the most important points in the face. These facial landmarks were used to calculate the nineteen facial ratios used in this analysis.  Figure 2 illustrates the facial landmarks (Figure 2(b)) for the input sample image (Figure 2(a)).

#### 2.1.2. Facial Feature Set Extraction

The facial beauty rating for all the 5500 images available with the dataset was used as a label for the nineteen ratios. These ratios are used as input to the model for the prediction of facial beauty. The basic premise of the feature set is that certain proportions of the face should follow defined ratios. Here, 14 golden ratios and five symmetry values have been used. A total of 19 values are referred to as the feature set (FS). To assess facial beauty based on facial proportion features, 19 ratios in the FS are analyzed. A detailed description of the various ratios in the FS is given in Tables 1 and 2. In Tables 1 and 2, *d*(*m*, *n*) refers to the Euclidean distance between landmarks *m* and *n*. The ratio values in the FS were different so normalization had to be performed on the ratios.

**Figure 3:**
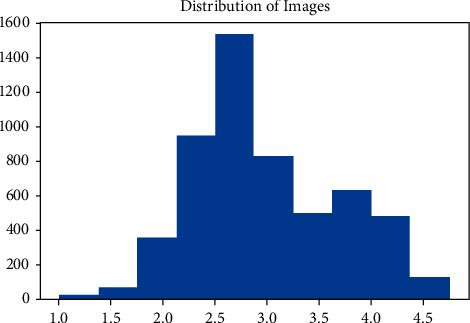
Distribution of beauty scores in the dataset.

In the Golden Ratios, attractive faces should have a ratio of 1.618 and in Symmetry Ratios, attractive faces should have a ratio of 1. So, *z*-score normalization and linear scaling are used to normalize the ratio values into an interval of [0, 1]. The normalization formula is given by the following:

(1) $$\begin{array}{c} \text{zi}=\frac{\text{si}-\text{mean}\left(S\right)}{\text{std}\left(S\right)},\\ z_{i}^{\prime }=\text{lb}+\frac{\text{zi}-\min\left(Z\right)}{\max\left(Z\right)-\min\left(Z\right)}\times \left(ub-lb\right), \end{array}$$

where si and zi denote the i^th^ original and normalized score values, respectively, mean()() and std()() denote the mean and standard deviation of the FS, lb and ub denote the lower bound (zero) and upper bound (1.618) of a target score range, and min()() and max()() denote the minimum and maximum values of a given score set, respectively.  Figure 3 shows the score distribution for each category in the dataset, namely Asian Male/Female and Caucasian Male/Female. The *X*-axis represents the score and the *Y*-axis represents the number of images.

#### 2.1.3. Secondary Feature Set Extraction

From previous literature, it is obvious that only facial landmarks and facial ratios cannot be used to provide good results while predicting facial beauty. Facial landmarks can only provide limited information regarding facial beauty. Also, it is known that humans decide beauty based on other characteristics like face color, shape, texture, etc. AL Jones [28] analyzed the effect that facial color had on the perception of facial beauty. Their study also concluded that better facial health, i.e., clear skin, reduced abnormalities, etc., positively correlated to higher attractiveness. Face shape also corresponds to attractiveness [23, 29], as shown in Jones and Zhao et al. The studies show that a more narrow face shape with sharp features corresponds to higher beauty as compared to a round face. Facial textures are also shown to correlate to higher beauty standards, as shown in Tan et al., [30]. The combination of facial textures and color provides more information about facial beauty than facial landmarks. This study aims to use facial shape, textures, and colors to predict facial beauty and infer the performance and contribution of features towards the performance of the model. These features are called as Secondary Feature Set or (SFS) in this study, and the types of features extracted are as follows:

- Texture Features GLCM Features (Correlation, Contrast, Energy, and Homogeneity)
- Shape Features Hu's Seven Invariant Moments
- Color Features Color Histograms in HSV Color Space

GLCM or Gray Level Covariance Matrix is also known as the Gray Level Spatial Dependence Matrix, which learns about the texture of an image by calculating the frequency of pixel pairs with certain values in a spatial relationship that occurs in an image. Various statistical measures are then extracted from this matrix which provides textural information of the image. GLCM features are extracted for this study as they provide good information regarding the spatial relationships in the image. The statistical descriptors and their description are provided in Table 3. Each statistic returns a single feature value for an image and the four features of Correlation, Contrast, Energy, and Homogeneity make up a feature vector that is concatenated with the best performing FS's given in the facial feature set extraction section.

Hu's Moments [31] or Hu's invariant moments are a set of 7 numbers calculated using central moments that are invariant to image transformations. The first 6 moments have been proved to be invariant to translation, scale and rotation, and reflection. While the 7th moment's sign changes for image reflection. The 7 moments are calculated by the below equations:

(2) $$\begin{array}{c} h_{0}=\eta_{20}+\eta_{02},\\ h_{1}={\left(\eta_{20}-\eta_{02}\right)}^{2}+4\eta_{11}^{2},\\ h_{2}={\left(\eta_{30}-3\eta_{12}\right)}^{2}+{\left(3\eta_{21}-\eta_{02}\right)}^{2},\\ h_{3}={\left(\eta_{30}+\eta_{12}\right)}^{2}+{\left(\eta_{21}+\eta_{03}\right)}^{2},\\ h_{4}=\left(\eta_{30}-3\eta_{12}\right)\left(\eta_{30}+\eta_{12}\right)\left[{\left(\eta_{30}+\eta_{12}\right)}^{2}-3{\left(\eta_{21}+\eta_{03}\right)}^{2}\right]+\left(3\eta_{21}-\eta_{03}\right)\left[3{\left(\eta_{30}+\eta_{12}\right)}^{2}-{\left(\eta_{21}+\eta_{03}\right)}^{2}\right],\\ h_{5}=\left(\eta_{20}-3\eta_{03}\right)\left[{\left(\eta_{30}+\eta_{12}\right)}^{2}-{\left(\eta_{21}+\eta_{03}\right)}^{2}+4\eta_{11}\left(\eta_{30}+3\eta_{12}\right)\left(\eta_{21}+\eta_{03}\right)\right],\\ h_{6}=\left(3\eta_{21}-\eta_{03}\right)\left(\eta_{30}+\eta_{12}\right)\left[{\left(\eta_{30}+\eta_{12}\right)}^{2}-3{\left(\eta_{21}+\eta_{03}\right)}^{2}\right]-\left(\eta_{30}-3\eta_{12}\right)\left(\eta_{21}+\eta_{03}\right){\left[3{\left(\eta_{30}+\eta_{12}\right)}^{2}-\left(\eta_{21}+\eta_{03}\right)\right]}^{2}. \end{array}$$

**Figure 4:**
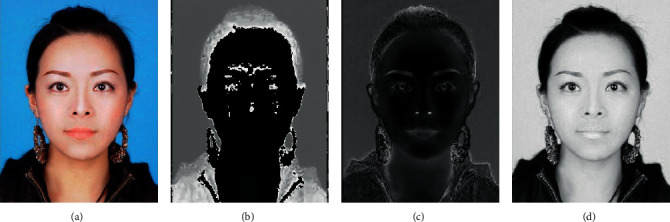
Sample image and its facial landmarks in various planes. (a) Original image. (b) Hue plane. (c) Saturation plane. (d) Value plane.

To calculate the facial color of the image, it is required to think about how an average person views a face. The HSV color space is more intuitive to how people experience color than the RGB color space [32]. As hue (*H*) varies from 0 to 1.0, the corresponding colors vary from red, through yellow, green, cyan, blue, and magenta, back to red. As saturation (*S*) varies from 0 to 1.0, the corresponding colors (hues) vary from unsaturated (shades of gray) to fully saturated (no white component). As value (*V*), or brightness, varies from 0 to 1.0, the corresponding colors become increasingly brighter. The sample image (Figure 4(a)) in Hue plane (Figure 4(b)), saturation plane (Figure 4(c)), and value plane (Figure 4(d)) are shown in Figure 4. With RGB, the color will have values like (0.5, 0.5, 0.25), whereas for HSV, it will be (30°, √3/4, 0.5). HSV is best used when a user is selecting a color interactively. It is usually much easier for a user to get the desired color as compared to using RGB [33].

#### 2.1.4. Models Used for Prediction

In this study, the FS is used as input to the models and the corresponding scores as the labels. Also, four well-known regression models, Linear Regression (LR), K-Nearest Neighbors (KNN), Random Forest (RF), and Artificial Neural Network (ANN), were used for prediction. A small description for each model is given below.

### 2.2. Linear Regression

Linear Regression is a statistical technique that uses several explanatory variables to predict the outcome of a response variable. It sets up a relationship between input variables and target variables which is represented by the following equation:

(3) $$\begin{array}{c} y_{i}=\beta_{0}+\beta_{1}x_{i1}+\beta_{2}x_{i2}+\cdots +\beta_{p}x_{ip}+\varepsilon , \end{array}$$

where *i* is the number of observations, *y*_*i*_ is the target variable, *x*_*i*_ is the input variable, *β*_0_ is the *y*-intercept, *β*_*p*_ is the coefficient for each input variable, and *ε* is the error term.

#### 2.2.1. Random Forest

Random forest is a Supervised Learning algorithm that uses ensemble learning methods for classification and regression. Random forest is a bagging technique and not a boosting technique. The trees in random forests are run in parallel. There is no interaction between these trees while building the trees. It operates by constructing a multitude of decision trees at training time and outputting the class that is the mode of the classes (classification) or mean prediction (regression) of the individual trees. A random forest is a meta-estimator (i.e., it combines the result of multiple predictions) which aggregates many decision trees, with some helpful modifications:

- The number of features that can be split at each node is limited to some hyperparameter. This ensures that the model does not rely too heavily on any individual feature and makes fair use of all potentially predictive features.
- Each tree draws a random sample from the original data set when generating its splits, adding a further element of randomness that prevents overfitting.

The above modifications help prevent the trees from being too highly correlated.

### 2.3. K-Nearest Neighbor (K-NN)

kNN falls is a lazy learning method, which means that there is no explicit training phase before classification. The Euclidean distance formula and probability formula of the kNN method are given in the following equations, respectively:

(4) $$\begin{array}{c} d\left(x,x{}^{\prime }\right)=\sqrt{{\left(x_{1}-x_{1}^{\prime }\right)}^{2}+{\left(x_{2}-x_{2}^{\prime }\right)}^{2}+\cdots +{\left(x_{n}-x_{n}^{\prime }\right)}^{2}}, \end{array}$$

(5) $$\begin{array}{c} P\left(y=j|X=x\right)=\frac{1}{K}\sum_{i\in A}I\left(y^{\left(i\right)}=j\right), \end{array}$$

where *A* is the particular class/set, unseen observation *x* and a similarity metric *d* and *K* is an arbitrary integer. *A* weighted average of the *K*-nearest neighbors were used, where the weight was decided by the Euclidean distance of the *K* closest training samples. The number of clusters is set to [12, 22].

### 2.4. Artificial Neural Network

In ANN regression, a multilayer perceptron (MLP) is applied which is composed of an input layer, hidden layers, and an output layer. Each layer has one or more neurons directionally linked with the neurons from the previous and next layers. A sigmoid function was applied as the activation function to compute the output of the hidden layer in each neuron. Artificial Neural Networks have been used to predict facial beauty with much success, as shown in [34].

### 2.5. Metrics Used for Prediction

To measure the model performance and calculate the error generated by the models, four metrics are used. Mean Absolute Error (MAE), Mean Squared Error (MSE), *R*^2^ Score, and Pearson's Correlation Coefficient (PC) [35] are provided in Table 4.

### 2.6. Experimental Setup

The dataset was split into training data (80%) and testing data (20%). All the experiments were run on an Intel i3 Processor with 12 GB of RAM using Python programming language. Two ablation studies were performed. One was to find the best performing FS out of all the primary features containing facial landmarks. The other ablation study was done to find out the performance variations amongst the concatenated SFS. The experimental results, along with the corresponding graphs, are explained in the results and discussion section.

## 3. Results and Discussion

In our study on facial beauty prediction, we used facial landmarks as a base feature set for extracting facial features. From the facial landmarks, we calculate 19 facial ratios that are used for predicting the beauty of the facial image (explained in Appendix A). It is observed that extracting and employing more features does not improve the performance of the proposed model. Further, these 19 facial ratios holistically describe the facial landmarks of the face, which indeed predict the symmetry and quantify the associated beauty [36, 37].

The analysis compared the performance of computer models against human ratings and examined the performance of each model.  Table 5 shows the performance of each model concerning the correlation and error metrics.

In Table 5, each model has a relatively high correlation with human ratings and low errors for each of their corresponding best performing FS.

**Figure 5:**
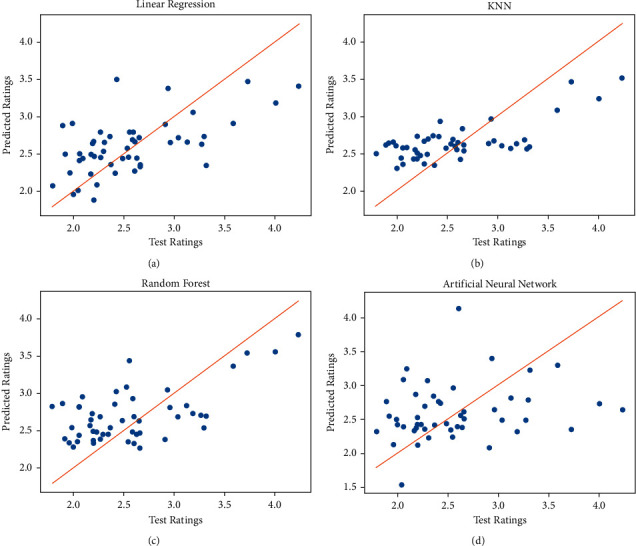
Regression curves for the best performing FS of each model. (a) Regression Curve for LR. (b) Regression curve for KNN. (c) Regression curve for RF. (d) Regression curve for ANN.

In Figure 5, the *x*-axis represents human scores on a scale of 0–5 and the *y*-axis represents the computer-predicted scores in the same range. KNN has the highest correlation values with the lowest error. ANN has a higher correlation than LR, but LR has comparatively lower error than ANN. Also, ANN is much more scattered than LR. The red line in the graphs shows the regression fit of the data. ANN is most similar to the ideal case. The second similar measure is LR, with the least predictive measures being KNN and RF. Therefore, while KNN and RF can correlate much more with the human values, ANN and LR are better models for fitting the data.

**Figure 6:**
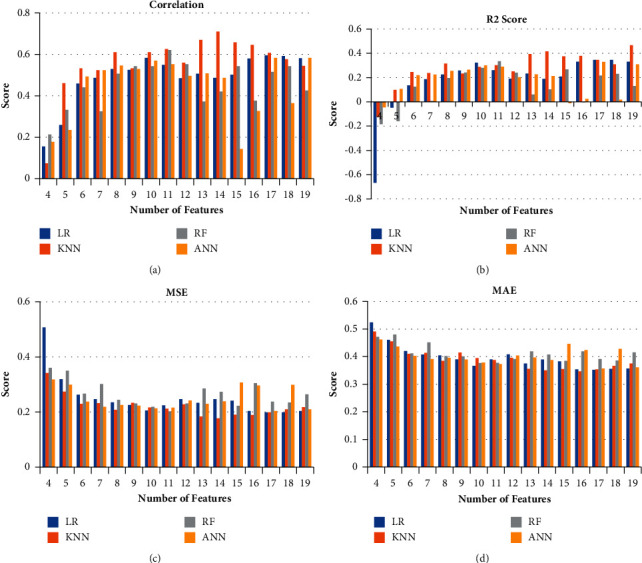
Ablation study of the metrics of FS for the various models. (a) Ablation Study of Correlation for LR, KNN, RF, and ANN. (b) Ablation studies of R2 Score for LR, KNN, RF, and ANN. (c) Ablation studies of MSE for LR, KNN, RF, and ANN. (d) Ablation studies of MAE for LR, KNN, RF, and ANN.

Even though Table 6 provides information about the best performing FS, Figure 6 shows us how each model learns from each feature in the FS and how each feature contributes to the learning process. The *X*-axis in Figure 6 represents the number of features used in the ablation study, and the *Y*-axis represents the Score for each metric. The *X*-axis starts from 4 because the metrics for features 3 and below were very poor, and hence it was decided not to be added in the graph as it would be negligible in comparing the performance of the models. It is trivial from Figure 6(b) when the number of features is less (for 5 and 6 in *X*-axis), the *R*^2^ is in a negative trend which indicates that the fit with regression curve is worse. Meanwhile, by increasing the number of features, the *R*^2^ values are improved significantly.

The nineteen features in the FS are ratios from Symmetry and Golden Ratios. From previous studies, it is known that Golden Ratios perform better than Symmetry Ratios. This phenomenon is proved when looking at the above tables. The best performing FS is obtained from the initial removal of Symmetry Ratios and the metrics decrease once the Golden Ratios are removed. Next, the ablation studies for the SFS have been performed. Tables 6 and 7 show the ablation studies performed for each model.

It is observed from Tables 6 and 7, the fusion of Texture, Color, and Shape features as the SFS along with the ratio features improves the overall performance of the model. The highest correlation is achieved by KNN, followed by RF. The least correlation is obtained through LR and ANN. In every model, each feature contributes differently to the performance of the model. Since the KNN model has achieved the highest correlation values and lowest errors, it is considered as the ideal model for facial feature analysis, and the obtained results are given in Table 8 (images and scores of few best and moderate prediction results).

From the above-obtained results, it is clear that our technique has avoided overfitting in our study through the following ways:

- The dataset used is from SCUT-FBP5500, which is a collection of 5500 frontal facial images. This dataset has been extensively used by many researchers, and there is no class imbalance, as shown from the histogram (Figure 3). The dataset has an equal number of images of each class in the training and testing set, and the target labels are normally distributed. The data used is enough, and since it is already balanced, no preprocessing or data augmentation was required.
- An Ablation study was conducted to find the best performing feature set and to find the optimal number of features which can be used to achieve the best performance. We avoided overfitting by removing features and limiting the maximum number of features to 19.

## 4. Conclusion

In this study, different computer models are analyzed to predict facial beauty using facial features like golden ratios, texture, shape, and color through Machine Learning. First, the models are analyzed and the performance on facial ratios is derived from Golden Ratios and Symmetry. Nineteen facial ratios were selected to represent the Feature Set (FS) from which the best performing features were derived by ablation study on each feature. From the best performing FS for each model, Texture, Color, and Shape Features were extracted from the dataset. This feature vector from secondary features (SFS) is fed to each model through an ablation study to measure the performance of each feature and observed the variation on performance metrics of the model. Experimental results showed that the beauty score obtained from KNN achieved the best metrics, followed by RF, LR, and ANN. The fusion of TCS Features with the FS to form the SFS performs the best, with the highest correlation being 78%. Therefore, a fusion of multiple feature types rather than a single feature type provided better performance than using one feature type. Also, heterogeneity of the feature vector increased the performance as compared to a homogenous feature vector. In addition, our analysis observed that Golden Ratios provided better information than Symmetry Ratios which was consistent with previous literature.

Our analysis has shown that human judgments regarding facial beauty are consistent with the facial ratios derived from painters, architects, etc. These ratios from Symmetry, Golden Ratios, Neoclassical canons, etc., correlate highly with human ratings. But, there does exist the element of variation, which exists as secondary features like sexual features, facial health, etc. Our analysis has shown that combining secondary features like facial health, texture, and shape with facial proportions allows a computer model to learn better and correlate much more with its human counterparts.

Overall, analysis has been limited with the facial beauty prediction of Asian and Caucasian males and females. Our future work will involve an analysis of facial beauty for different types of people. Alongside, our analysis will use more powerful algorithms to extract deep features and to use the same fusion technique on those features to achieve an even higher correlation and lower error. Also, it is aimed to increase the size of the dataset and variation to allow for better generalization. Another work is aimed to make a modular system that can rate beauty in real-time to allow for various beauty evaluation applications.

## Figures and Tables

**Figure 7:**
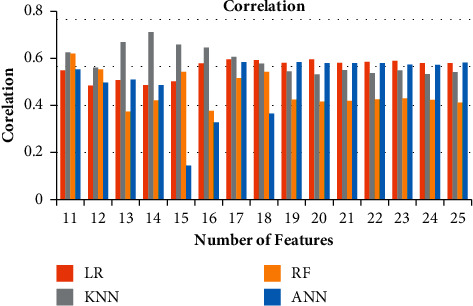
Ablation study of correlation for 11–25 features.

**Figure 8:**
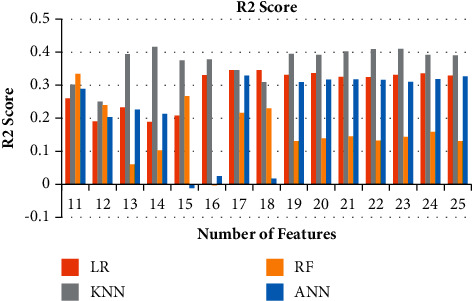
Ablation study of R2 Score for 11–25 features.

**Figure 9:**
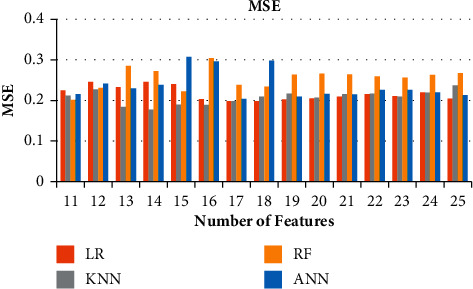
Ablation study of MSE for 11–25 features.

**Figure 10:**
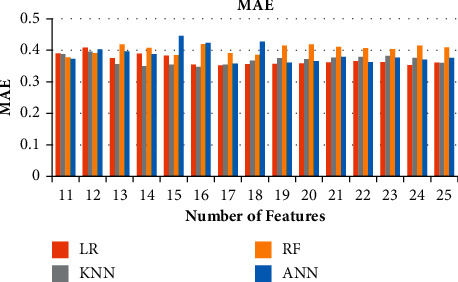
Ablation study of MAE for 11–25 features.

**Table 1:** Description of facial ratios used.

S. No.	Description	Ratio vector
1.	Under eyes/Interocular	*d*(49,57)/*d*(43,55)
2.	Under eyes/Nose width	*d*(49,57)/*d*(65,59)
3.	Mouth width/Interocular	*d*(80,87)/*d*(43,55)
4.	Upper lip-jaw/Interocular	*d*(77,12)/*d*(43,55)
5.	Upper lip-jaw/Nose width	*d*(77,12)/*d*(65,59)
6.	Interocular/Lip height	*d*(43,55)/*d*(77,83)
7.	Nose width/Interocular	*d*(65,69)/*d*(43,55)
8.	Nose width/Upper lip height	*d*(65,69)/*d*(77,84)/2
9.	Interocular/Nose mouth height	*d*(43,55)/*d*(67,77)
10.	Face top-eyebrows/Eyebrows-Nose	*d*(1, *d*(23,37)/2)/*d*(*d*(23,37)/2, 67)
11.	Eyebrows-nose/Nose-jaw	*d*(*d*(23,37)/2, 67)/*d*(67,12)
12.	Face top-eyebrows/Nose-Jaw	*d*(1, *d*(23,37)/2)/*d*(67,12)
13.	Interocular/Nose width	*d*(43,55)/*d*(65,69)
14.	Face height/Face width	*d*(1,12)/*d*(7,17)

**Table 2:** Description of Symmetry ratios used.

S. No.	Description	Ratio vector
1.	Lower eyebrow length	*d*(27,32)/*d*(38,42)
2.	Lower lip length	*d*(80,84)/*d*(74,84)
3.	Upper eyebrow	*d*(23, *d*(23,37)/2)/*d*(35, *d*(23,37)/2)
4.	Upper lip	*d*(80,77)/*d*(74,77)
5.	Nose	*d*(65,67)/*d*(69,67)

**Table 3:** Statistical descriptors for GLCM.

Statistic	Description	Justification	Formula
Contrast	This measure returns the intensity contrast between a pixel and its neighbor over the image.	This feature can help in measuring the number of local variations in the image.	∑_*i*,*j*_|*i* − *j*|^2^*p*(*i*, *j*)

Homogeneity	This measure measures the similarity between the diagonal elements of the GLCM and the distribution of elements in the GLCM	This measure helps in finding out the degree to which each pixel differs from the other.	∑_*i*,*j*_*p*(*i*, *j*)/1+|*i* − *j*|

Correlation	This measure returns the correlation between a pixel and its neighbor over the whole image.	This feature can help in measuring how much a pixel relates to the whole image.	∑_*i*,*j*_(*i* − *μi*)(*j* − *μj*)*p*(*i*, *j*)/*σ*_*i*_*σ*_*j*_

Energy	This measure measures the sum of squared elements in GLCM. It is also known as uniformity.	This measure helps in finding out the disorders in the texture of an image.	∑_*i*,*j*_*p*(*i*, *j*)^2^

**Table 4:** Various matrices used for prediction.

S. No	Metrics	Formula
1.	MAE	MAE=1/*n*∑_*i*=1_^*n*^|*x*_*i*_ − *x*|
2.	MSE	MSE=1/*n*∑_*i*=1_^*n*^|*x*_*i*_ − *x*|^2^
3.	*R* ^2^	$R^{2}=1-\sum_{i}{\left(y_{i}-y_{i}^{\prime }\right)}^{2}/\sum_{i}{\left(y_{i}-\bar{y_{i}}\right)}^{2}$
4.	PC	$\text{PC}=\frac{n\left({\sum xy}^{}\right)-\left({\sum x}^{}\right)\left({\sum y}^{}\right)}{\sqrt{\left[n\sum x^{2}-{\left({\sum x}^{}\right)}^{2}\right]\left[n\sum y^{2}-{\left({\sum y}^{}\right)}^{2}\right]}}$

**Table 5:** Best Performing FS for each model.

S. No	Model name	PC	*R*^2^ score	MAE	MSE
1.	LR	0.5811	0.3310	0.3570	0.2031
2.	KNN	0.7115	0.4162	0.3504	0.1773
3.	RF	0.6201	0.3344	0.3777	0.2019
4.	ANN	0.5838	0.3096	0.3610	0.2096

**Table 6:** Ablation study of PC and *R*^2^ score for various models

S. No.	Features used	PC of SFS for				*R*^2^ score of SFS for			
		LR	KNN	RF	ANN	LR	KNN	RF	ANN
1.	Without	0.58	0.71	0.62	0.58	0.33	0.41	0.33	0.30
**2.**	**T + C + S**	**0.65**	**0.78**	**0.70**	**0.62**	**0.40**	**0.48**	**0.40**	**0.36**
3.	*T* + *C*	0.64	0.76	0.68	0.60	0.39	0.46	0.38	0.35
4.	*T* + *S*	0.60	0.75	0.67	0.59	0.37	0.45	0.36	0.33
5.	*C* + *S*	0.62	0.74	0.67	0.61	0.38	0.45	0.37	0.34
6.	*T*	0.59	0.74	0.65	0.58	0.38	0.47	0.34	0.34
7.	*C*	0.58	0.72	0.63	0.59	0.35	0.42	0.37	0.32
8.	*S*	0.57	0.73	0.64	0.60	0.34	0.43	0.35	0.31

**Table 7:** Ablation study of MAE and MSE score for various models.

S. No.	Features used	MAE of SFS for				MSE score of SFS for			
		LR	KNN	RF	ANN	LR	KNN	RF	ANN
1.	Without	0.35	0.35	0.37	0.36	0.20	0.17	0.20	0.20
**2.**	**T + C + S**	**0.30**	**0.28**	**0.29**	**0.31**	**0.15**	**0.09**	**0.11**	**0.15**
3.	*T* + *C*	0.31	0.30	0.30	0.33	0.16	0.12	0.12	0.16
4.	*T* + *S*	0.32	0.32	0.31	0.35	0.18	0.11	0.12	0.17
5.	*C* + *S*	0.31	0.31	0.30	0.33	0.17	0.11	0.11	0.15
6.	*T*	0.33	0.31	0.35	0.35	0.16	0.15	0.15	0.18
7.	*C*	0.32	0.34	0.32	0.34	0.15	0.16	0.13	0.17
8.	*S*	0.34	0.33	0.334	0.33	0.19	0.14	0.17	0.18

**Table 8:** Results obtained from the study through concatenation of TCS Features with SFS.

Actual value	Predicted value	Image
4.7	4.405	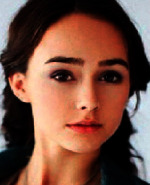
4.63	4.65	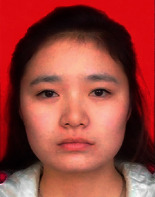
4.7	4.43	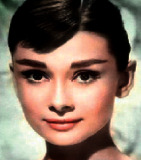
2.7	3.8	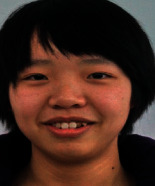
1.43	2.8	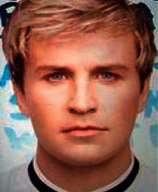

**Table 9:** Performance of each model according to correlation for 11–25 features.

S. No.	Number of features	LR	KNN	RF	ANN
1.	11	0.5492	0.625	**0.6201**	0.5527
2.	12	0.4842	0.5603	0.5535	0.4972
3.	13	0.5076	0.6694	0.3729	0.509
4.	14	0.4859	**0.7115**	0.4211	0.486
5.	15	0.5019	0.6587	0.542	0.1443
6.	16	0.5792	0.6458	0.3761	0.3275
7.	17	**0.5952**	0.6067	0.5158	0.5837
8.	18	0.5921	0.5773	0.5424	0.3648
9.	19	0.5811	0.5449	0.4248	**0.5838**
10.	20	0.5954	0.5321	0.4156	0.5801
11.	21	0.5806	0.5495	0.4187	0.5796
12.	22	0.5854	0.5369	0.4258	0.58
13.	23	0.5897	0.5485	0.4296	0.5732
14.	24	0.5801	0.5331	0.4235	0.5721
15.	25	0.58	0.541	0.4113	0.5821

**Table 10:** Performance of each model according to *R*^2^ Score for 11–25 features.

S. No.	Number of features	LR	KNN	RF	ANN
1.	11	0.2604	0.302	**0.3344**	0.2888
2.	12	0.1904	0.2509	0.2402	0.203
3.	13	0.2331	0.3945	0.0604	0.2264
4.	14	0.1892	**0.4162**	0.1029	0.213
5.	15	0.2082	0.3753	0.2676	-0.0114
6.	16	0.3303	0.3787	-0.003	0.0246
7.	17	**0.3459**	0.3462	0.2165	0.3288
8.	18	0.3457	0.309	0.2298	0.0178
9.	19	0.331	0.3952	0.1308	**0.3096**
10.	20	0.3369	0.3924	0.1395	0.3169
11.	21	0.3256	0.4021	0.1456	0.3178
12.	22	0.3248	0.4089	0.1324	0.316
13.	23	0.331	0.4097	0.1437	0.3099
14.	24	0.3358	0.3924	0.1587	0.3184
15.	25	0.3294	0.3910	0.1308	0.3269

**Table 11:** Performance of each model according to MSE for 11–25 features.

S. No.	Number of features	LR	KNN	RF	ANN
1.	11	0.2246	0.2119	**0.2019**	0.2159
2.	12	0.2458	0.2274	0.2307	0.242
3.	13	0.2329	0.1839	0.2853	0.2295
4.	14	0.2462	**0.1773**	0.2724	0.239
5.	15	0.2404	0.1897	0.2224	0.3072
6.	16	0.2033	0.1886	0.3046	0.2963
7.	17	**0.1986**	0.1985	0.2379	0.2038
8.	18	0.1987	0.2098	0.2339	0.2983
9.	19	0.2031	0.2171	0.2639	**0.2096**
10.	20	0.2056	0.2069	0.2658	0.2165
11.	21	0.2098	0.2154	0.2645	0.2148
12.	22	0.2154	0.2167	0.2594	0.2259
13.	23	0.2106	0.2098	0.2561	0.226
14.	24	0.2198	0.2192	0.2632	0.2197
15.	25	0.2046	0.2367	0.2674	0.213

**Table 12:** Performance of each model according to MAE for 11–25 features.

S. No.	Number of features	LR	KNN	RF	ANN
1.	11	0.3903	0.3876	**0.3777**	0.3727
2.	12	0.4083	0.3957	0.3914	0.4034
3.	13	0.3753	0.3558	0.4188	0.3964
4.	14	0.3897	**0.3504**	0.4079	0.3876
5.	15	0.3832	0.3546	0.3844	0.446
6.	16	0.3543	0.3468	0.4193	0.4236
7.	17	**0.3521**	0.3542	0.3913	0.3572
8.	18	0.3561	0.3667	0.3858	0.4279
9.	19	0.357	0.3749	0.4153	**0.361**
10.	20	0.3589	0.3725	0.4188	0.3654
11.	21	0.3621	0.3763	0.4103	0.3789
12.	22	0.3652	0.3792	0.4069	0.3625
13.	23	0.3624	0.3824	0.4037	0.3764
14.	24	0.3527	0.3761	0.4152	0.371
15.	25	0.361	0.3601	0.4092	0.3762

## Data Availability

The data set used for this study is openly available and the details are mentioned in the article. Data sets used in this study can be found on the website https://github.com/HCIILAB/SCUT-FBP5500-Database-Release/, and the codes are available openly in the repository https://github.com/IyerOnFyer/Facial-Beauty-Prediction.git.

## Appendix

### A

The results from the Ablation study for each model to predict the facial beauty are shown as Tables and Figures that experimentally verify that 19 facial ratios are indeed present the best performance.

As can be seen from Tables 9–Table 12 and Figures 7–Figure 10 , the performance of each model after 19 features does not improve in any significant manner. Due to this reason, model performance was calculated until 19 features. As mentioned in the study, the below 4 features were not considered as the performance was poor, which can be seen from negative performance metrics.
